# Digital health technologies for myopia care: a systematic review

**DOI:** 10.3389/fpubh.2026.1939923

**Published:** 2026-10-16

**Authors:** Haofei Xu, Liuqing He, Zhou Pan, Xuejing Lu

**Affiliations:** 1 Eye School, Chengdu University of Traditional Chinese Medicine, Chengdu, Sichuan, China; 2 Sichuan Provincial Key Laboratory of Traditional Chinese Medicine for Ophthalmic Health, Chengdu, Sichuan, China; 3 Retinal Image Technology and Chronic Vascular Disease Prevention & Control and Collaborative Innovation Center, Chengdu, Sichuan, China

**Keywords:** digital health technologies, myopia, smartphone, wearable device, website

## Abstract

**Purpose:**

Review the application and efficacy of digital health technologies (DHTs) in myopia care.

**Methods:**

This systematic review is registered on PROSPERO (CRD42024626798). Using appropriate medical subject headings and keywords, the authors searched multiple databases (PubMed, Embase, Cochrane, China National Knowledge Infrastructure, Wanfang data and VIP Database) to identify relevant studies published between 1 January 2000 and 1 December 2025. After duplicate removal, the titles and abstracts were screened, followed by full-text eligibility assessment and quality evaluation. Risk of bias was assessed by the Study Quality Assessment Tools. The quality of evidence was graded using the GRADEpro system.

**Results:**

Thirty-three articles with 26,651 patients were finally included in this review. This review included 5 cohort studies, 1 cross-over study, 15 cross-sectional studies, 2 non-RCTs, 5 RCTs, and 5 self-controlled studies. These 33 studies managed myopia by visual and refractive monitoring (*n* = 10), environmental and behavioral assessment (*n* = 7), visual and refractive improvement (*n* = 7), disease diagnosis (*n* = 6) and behavioral interventions (*n* = 3).

**Conclusion:**

Preliminary evidence suggests that DHTs may have potential values in myopic detection, treatment and management. However, the current evidence base is limited by small sample sizes, high heterogeneity, and very low certainty across all domains. Well-designed RCTs are needed to confirm their effectiveness. Given the predominance of evidence from East Asia, applicability to other regions remains uncertain.

## 1 Introduction

Myopia develops in the early stage, causing blurred vision when viewing distant objects (1). The number of people with myopia is projected to increase to 4,758 million (49.8% of the global population) by 2050 all around the world. Meanwhile, the number in high myopia will increase to 938 million by 2050 (9.8% of the global population) (2). Given the current rapid rise in the prevalence of myopia (especially in younger cohorts), environmental factors (decreased time outdoors and increased near work activities) are considered important risk factors for myopia (3). This creates the urgent need for comprehensive myopia care including detection, treatment and management (4).

Digital health technology (DHT) refers to devices and digital tools used for the generation and analysis of health data, which includes health data analysis, mobile health applications, wearable devices, clinical decision support, lifestyle monitoring, virtual reality, artificial intelligence (AI) (5, 6). DHTs have attracted growing attention from the society, especially in lower-income countries, where digital technology has provided a solution for overcoming geographical inaccessibility of health care (7). As a key population for myopia prevention and control, adolescents are also active users of digital technologies, providing a broad user base for myopia care. To evaluate the application of DHTs, we conducted a systematic review of the literature to examine whether DHTs are available and effective for myopia care.

## 2 Methods

### 2.1 Data sources and search strategy

This systematic review is registered on PROSPERO (CRD42024626798) and based on the Preferred Reporting Items for Systematic reviews and Meta-Analyses (PRISMA) guidelines. The PRISMA checklist, search strategy and data extraction can be found in Supplementary Tables 2, 3. We searched the PubMed, Embase, Cochrane, China National Knowledge Infrastructure, Wanfang data and VIP Database for studies from January 1, 2000, to December 1, 2025, on digital health technologies for myopia care. This period was chosen because of the rapid development of emerging technologies, bringing new possibilities for DHTs to intervene in the development of myopia. Search terms include “myopia,” “artificial intelligence,” “digital,” “digital health,” “digital therapeutics,” “e-health,” “telehealth,” “telemedicine,” “m-health,” “mobile applications,” “wearable devices,” “internet,” “online systems,” “social media,” “virtual reality,” “smartphone,” “spectrum,” “website.” Grey literature, including dissertations, full-length conference papers, and patents, was adopted to ensure a comprehensive search. Zotero 7 was used for literature management and screening.

### 2.2 Inclusion and exclusion criteria

Two researchers independently reviewed articles based on pre-specified inclusion and exclusion criteria. Disagreements between the two reviewers were resolved through discussion with a third reviewer to reach a consensus decision. Inter-rater reliability was assessed using Cohen’s kappa coefficient. The research questions were formulated following the PICOS principle. The study design exclusion criteria were as follows: 1. Retrospective designs were excluded because they are prone to recall and recording bias. 2. Not full-length publication (conference abstract, note, letter). 3. Narrative reviews, systematic review or meta analysis (specific exclusion reasons for each article can be found in Supplementary Table 4). The inclusion criteria were as follows:

#### 2.2.1 Detection

Population: children or adults at risk of myopia; Intervention: DHTs related to the screening and diagnosis; Comparator: clinical reference standards; Outcomes: agreement (visual acuity, refractive error) and diagnostic accuracy (sensitivity, specificity).

#### 2.2.2 Treatment

Population: individuals with myopia; Intervention: DHTs related to the treatment; Comparator: No intervention (usual care), sham or placebo intervention, alternative methods, baseline; Outcomes: visual acuity, refractive error.

#### 2.2.3 Management

Population: children or adults with or at risk of myopia; Intervention: DHTs related to the monitoring and management; Comparator: No intervention (usual care), sham or placebo intervention, alternative methods, baseline; Outcomes: behavioral and environmental modification.

#### 2.2.4 Operational definition

For this review, we defined digital health technologies as digital tools (applications, website tools, wearable devices, VR, and AI) used for myopia detection, treatment, or management. Eligible technologies were those targeting individuals with myopia or at risk of myopia, including those with myopia-related conditions, and reporting at least one quantitative outcome on myopia detection, treatment, or management. We excluded technologies serving solely as data storage or administrative tools without direct patient interaction or clinical assessment, as well as non-digital interventions without a digital component.

### 2.3 Data extraction

The extracted data include: 1. Study design and characteristics: first author, year, country, study design, outcomes; 2. Characteristics of the digital intervention: name, type (mobile applications, website), device (smartphone, wearable devices, computer), theoretical approach, follow-up duration, primary outcome measure (secondary); 3. Sample characteristics: sample size, age, sex; 4. Results of the intervention: effectiveness; 5. Effect estimates and precision: reported effect, 95% confidence intervals, direction of effect, and *p*-value (the detailed results of the data extraction can be found in Supplementary Table 3).

### 2.4 Assessment of the study quality

The quality of the articles was independently assessed by two evaluators using the Study Quality Assessment Tools (the detailed results of the quality assessment are presented in Supplementary Table 1) (8).

### 2.5 GRADE rating of the quality of evidence

We used the GRADEpro tool to assess the certainty of evidence for the primary outcomes, considering study design, risk of bias, inconsistency, indirectness, and imprecision. The certainty of evidence for each outcome measure was classified as high, moderate, low, or very low (the GRADE assessments are presented in Table 1).

**Table 1:** Vote-counting results for digital health technologies in myopia care.

Clinical categories	Outcome types	Studies reporting (*n*)	Beneficial effects/overestimate	No clear difference/agreement	Poor effects/underestimate	Quality of evidence
Detection	Disease diagnosis	6	6	0	0	⊕◯◯◯
	Visual acuity	6	0	0	6	⊕◯◯◯
	Refractive error	7	4	0	3	⊕◯◯◯
Treatment	Visual and refractive improvement	7	7	0	0	⊕◯◯◯
Management	Behavioral interventions	3	3	0	0	⊕◯◯◯

AUC, area under the receiver operating characteristic curve. ⊕◯◯◯, very low quality of evidence.

For agreement studies against clinical standards: beneficial effects was sensitivity/specificity ≥ 0.80, AUC ≥ 0.80; no clear difference/agreement: was 0.80 > sensitivity/specificity ≥0.50, 0.8 > AUC ≥ 0.50, or when opposite directions of deviation were observed in same measurement; poor effects was sensitivity/specificity <0.50, clinically meaningful systematic deviation without documented harm, and negative tool use directly associated with adverse outcomes (54–56).

### 2.6 Data synthesis

Meta-analysis was not performed owing to substantial clinical and methodological heterogeneity across the included studies, particularly with respect to study designs, types of digital health technologies, outcome measures, and measurement instruments (9). Following the Cochrane Handbook, a vote-counting synthesis based on direction of effect was adopted. Effects were classified as beneficial effects, no difference, or detrimental effects. This approach describes only the direction and consistency of effects across studies and does not estimate the magnitude or clinical importance of any benefit. For measurement studies, effects were classified as overestimate, no clear difference, or underestimate. The other studies, whose direction of effect could not be determined, a structured narrative synthesis was used instead of vote counting. In cases with multiple reported follow-up timepoints, end-of-intervention assessments were selected preferentially.

## 3 Results

### 3.1 Study selection

We retrieved 6,370 articles from the databases as of December 1, 2025. After removing 1,459 duplicates, 4,911 articles were retained. After screening the titles and abstracts of 4,911 records, we excluded 4,702 articles that failed to meet our predefined PICOS criteria, leaving 209 records eligible for full-text screening. A total of 205 articles were included in the full-text review, excluding 4 articles for which full text could not be found. Based on the inclusion and exclusion criteria, we excluded 172 articles. In conclusion, a total of 33 articles were included in this review, and all of them underwent quality assessment (Figure 1). The Cohen’s kappa value was 0.75 for the title/abstract screening (4,911 records) and 0.82 for the full-text screening (205 records).

**Figure 1:**
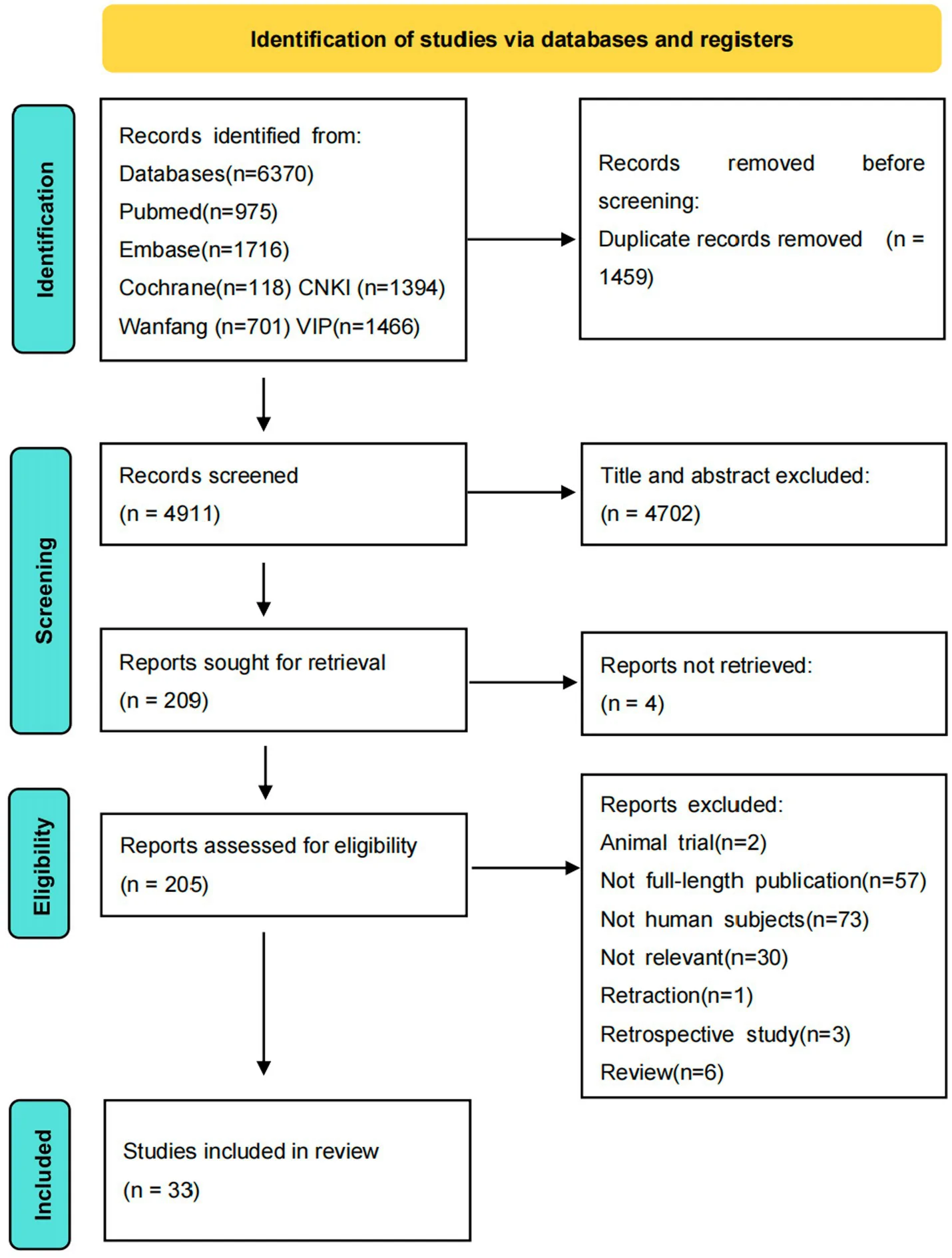
Flowchart of the screening process.

### 3.2 Study characteristics

Overall, thirty-three articles with 26,651 patients were finally included in this review. This review included 5 cohort studies, 1 cross-over study, 15 cross-sectional studies, 2 non-RCTs, 5 RCTs, and 5 self-controlled studies. These 33 studies managed myopia by visual and refractive monitoring (*n* = 10), environmental and behavioral assessment (*n* = 7), visual and refractive improvement (*n* = 7), disease diagnosis (*n* = 6), behavioral interventions (*n* = 3). The studies were conducted in North America (*n* = 1), Africa (*n* = 1), Europe (*n* = 4), Asia (*n* = 26) and Asia/North America (*n* = 1).

The DHTs utilize AI, systems, websites, software, applications, virtual reality, and other digital methods. They mainly rely on modern smart devices such as smartphones, computers, digital cameras, tablets and wearable devices. In total, 21 studies were rated as Good and 12 as Fair in the quality assessment (Cohen’s kappa = 0.77) (see Table 2).

**Table 2:** Summary of the articles included in the review (*n* = 33).

Characteristics	*n*	%
Study design		
Cross-sectional study	15	45.45%
Cohort study	5	15.15%
Self-controlled study	5	15.15%
Randomized clinical trial	5	15.15%
Cross-over study	1	3.03%
Non-randomized controlled trial	2	6.06%
Region (continent)		
Asia	26	78.79%
Europe	4	12.12%
North America	1	3.03%
Africa	1	3.03%
Asia/North America	1	3.03%
Publication year		
2021–2025	22	66.67%
2016–2020	10	30.30%
2010–2015	1	3.03%
Main function		
Visual and refractive monitoring	10	30.30%
Environmental and behavioral assessment	7	21.21%
Visual and refractive improvement	7	21.21%
Disease diagnosis	6	18.18%
Behavioral interventions	3	9.09%

### 3.3 Detection

#### 3.3.1 Disease diagnosis

There are six studies applying digital approaches to the diagnosis of diseases (10–15). All six studies adopted sensitivity and specificity as primary outcome measures for disease diagnosis, ranging from 0.76 to 1.00. Two studies used smartphone flashes and cameras to identify eye diseases (10, 13). One study assembled the lighting conversion unit to the digital camera and calculated diopter and dark zone height (12). The other 2 studies both constructed deep learning or artificial intelligence models to enable batch interpretation of multiple refractive errors or fundus lesions (11, 14). Li et al. applied tablet-based vision screener and get better sensitivity and specificity in older generation (15).

#### 3.3.2 Visual and refractive monitoring

In total, ten studies focused on vision monitoring by system, application, software, and website (16–25). Three studies adopted only visual acuity (VA) as the outcome measure (22–24), four used refractive error (RE) alone (17–19, 21), and the remaining three incorporated both VA and RE as outcome measures simultaneously (16, 20, 25). Smartphones were the most common devices, used in all 10 studies. Two studies required cellphones in conjunction with the Netra wearable device (16, 17). However, both of them reported Netra produced worse outcomes in refractive error, which could yield a myopic overcorrection compared to subjective refraction. Hasrod and Rubin (17) found that myopic overcorrection of Netra can be improved with cycloplegia. The Netra results were refined by a refraction-trained practitioner using the Netropter, a handheld phoropter included with the EyeNetra system (16). No statistically significant difference was observed between the refined Netra measurements and professional subjective refraction. A total of two studies using the Easee web-based testing platform required participants to operate smartphones alongside computers or tablets, with visual acuity and refractive error set as the outcome measures (20, 25). These two studies demonstrated that the Easee device achieved favorable agreement for VA measurements within the 95% confidence interval, whereas contradictory findings were observed for refractive error assessments. Further studies are needed to identify the factors contributing to Easee’s overestimation or underestimation of refractive error. Claessens et al. (20) also reported a remarkably proportional bias in Easee, with a relative underperformance when VA declined. In China, two studies used the Wise Eye System for visual acuity screening, which enables automatic adjustment of optotype size and brightness (22, 24). No statistical difference was detected in VA measurements between the Wise Eye System and the reference standard method.

### 3.4 Treatment

#### 3.4.1 Visual and refractive improvement

Overall, seven studies applied DHTs to improve VA or reduce refractive error (26–32). six studies employed VA as the outcome measure, while one study relied on RE (28). Virtual reality technology was used to do virtual training in four articles. Following VR training, uncorrected visual acuity improved across all five studies, with follow-up durations ranging from 10 days to 1 year (26, 27, 29–31).

Notably, two studies adopted wearable VR devices for the treatment of myopic anisometropic amblyopia. The intervention yielded improvements not only in best-corrected visual acuity, but also in stereopsis, refractive error, P100 latency and P1 amplitude (26, 27). Nevertheless, further trials are still required to verify the efficacy of the Virtual Reality Visual Perception Platform in children with simple myopia. One study focused on ocular muscles training (32). The system guides users to perform regular eye movements by dynamic icons and provides interactive training combining eye movements with manual operations. Shao et al. prevented and controlled children myopia by digital intelligent modulation far-infrared hot moxibustion technology, which combined Traditional Chinese Medicine and modern digital technology (28). It operates by emitting radiation at a wavelength of approximately 10 μm to mimic the beneficial ingredients of natural sunlight. Meanwhile, it delivers moxibustion therapy to periocular acupoints. The constant-temperature electric heating relaxes tense ciliary muscles and boosts local blood circulation.

### 3.5 Management

#### 3.5.1 Environmental and behavioral assessment

Seven studies conducted environmental monitoring for myopia, using wearable devices (*n* = 4) (33–36), smartwatches (*n* = 2) (37, 38), and smartphones (*n* = 1, 39).

Due to the diversity of environmental risk factors for myopia, consistent outcome measures were not adopted across the seven studies, which mainly included the duration or distance of near work (*n* = 5) (33–36, 39), outdoor time (*n* = 6) (34–39), and light intensity (*n* = 5) (33, 34, 36–38). Two studies demonstrated that continuous near-work duration was associated with myopia onset (36, 39). Li et al. (36) applied the Clouclip to quantify ocular behaviors among school-aged children. The myopic group presented significantly longer daily total near-work time (237.06 ± 85.82 min) and single-session near-work duration (79.28 ± 27.31 min) compared to the non-myopic group (140.62 ± 53.89 min and 36.06 ± 12.97 min, respectively). Enthoven et al. (39) adopted a myopia monitoring application to track eye-use behaviors. Continuous use consisted of 6.42 ± 4.36 uninterrupted 20-min sessions was significantly correlated with spherical equivalent refraction (SER) and the axial length/corneal radius (AL:CR) ratio. In addition, a working distance of 40 cm was linked to adverse myopia-related effects (33). One study emphasized enough outdoor time (at least 15 min) is benefit against myopic shift (38). Two studies found that appropriate light intensity took positive effects to prevent myopia (33, 38).

#### 3.5.2 Behavioral interventions

Overall, three studies were dedicated to correcting eye usage habits, with follow-up durations ranging from 15 to 83 days (40–42). Tablet (*n* = 2) and wearable device (*n* = 1) were applied to these studies. All of the studies regarded near-work distance as an important part of outcome measures. Two studies triggered an alarm once the viewing distance dropped below 30 cm (40, 41), whereas one study partially dimmed the tablet screen when the face-device distance was less than 40 cm (42). Wang utilized an AI-powered tablet that could not only correct improper near-work behaviors but also identify abnormal viewing postures (when the head roll angle exceeded ±30° for over 12 s) and send out corresponding alerts (40) (see Table 3).

**Table 3:** Characteristics of the included studies.

Citation	First author	Year	Outcomes	Main function	Type	Device	Primary outcome measure(secondary)
(10)	Shuoxin Ma	2020	For myopia detection, the sensitivity and specificity were 0.83 and 1.00, respectively.	Disease diagnosis	Application	Smartphone	Sensitivity and specificity of the application
(33)	Lei Li	2020	Around 40 cm of working distance and 6,300 lux of light intensity are linked to adverse effects related to myopia.	Environmental and behavioral assessment	Sensor	Wearable device	Working distance, eye-level light intensity, spherical equivalent refraction
(34)	Wu Ziyun	2023	Notable correlations existed between measured values and set values when considering working distance and time, sitting head tilt angle, indoor light intensity, outdoor time, face-to-screen time.	Environmental and behavioral assessment	AI system	Wearable device	Working distance, head tilt angle, indoor light intensity, near-work time, average continuous near-work time, outdoor activity time, time watching computers, and time watching phones
(40)	Jifang Wang	2024	AI notifications rectified the majority of incorrect viewing habits related to both viewing distance and viewing posture.	Behavioral interventions	AI system	Tablet	Usage time, near-work distance, light intensity, viewing content, blinking rate
(41)	Yingpin Cao	2020	The time ratio of near-work activity <30cm to the total <60cm and the frequency of continuous near-work were decreased after the intervention.	Behavioral interventions	Application, cloud platform	Wearable device	Near-work time and distance
(11)	Qin Shu	2024	The model for myopia identification achieved a sensitivity of 0.84 and a specificity of 0.76.	Disease diagnosis	AI system	Smartphone	Sensitivity, specificity, accuracy, AUC and F1-score of the AI model
(12)	Bin Wang	2011	The specificity of the equipment for hyperopia, myopia and astigmatism was 96.46%,95.29%,96.75%, and the sensitivity was 82.76%, 93.33%, 84.78%.	Disease diagnosis	System	Pocket-sized digital camera	Sensitivity and specificity of the pocket-sized digital camera
(26)	Wei Shigang	2019	After virtual training, BCVA in the intervention group improved from 0.48 ± 0.30 to 0.77 ± 0.27, with improvement in fine stereopsis.	Visual and Refractive Improvement	System	VR wearable device	VA (visual function, fine stereopsis)
(27)	Liu Zongshun	2023	After visual training, the intervention group showed improved BCVA (from 0.56 ± 0.03 to 0.82 ± 0.09) and near visual acuity, as well as decreased stereoscopic acuity, diopter, P100 latency and P1 amplitude.	Visual and Refractive Improvement	System	VR wearable device	VA (near stereopsis, contrast sensitivity, stereoscopic acuity, and visual evoked potentials)
(28)	Shao Ligong	2022	At the 24th and 48th weeks after intervention, the diopter, axial length, corneal curvature and intraocular pressure in the experimental group were significantly improved (*p* < 0.05).	Visual and Refractive Improvement	Embedded program	Hot Moxibustion Technology	Refractive error, axial length, corneal curvature and intraocular pressure
(16)	Benoit Tousignant	2020	Unassisted Netra had a median myopic overcorrection of 0.60 D compared to professional subjective refraction.	Visual and refractive monitoring	System	Smartphone and Netra device	VA, refractive error and Likert scale
(17)	Nabeela Hasrod	2022	On average, NETRA without cycloplegia exhibited a myopic shift of around 1.25 D.	Visual and refractive monitoring	Application	Smartphone and Netra device	Refractive error
(18)	Qiu kaikai	2024	There were no statistically differences in the measurements of spherical equivalent, cylinder, axis, and pupillary distance between software and the computerized autorefractor (*p* > 0.05).	Visual and refractive monitoring	Software	Smartphone	Refractive error
(37)	Pavan K. Verkicharla	2017	On average, children spent about 1 h in light levels greater than 1,000 lux on weekdays and 1.3 h on weekends. The mean light intensity level was 731 ± 501 lux and the maximum level was 31,669 ± 32,415 lux.	Environmental and behavioral assessment	Application	Smartwatch	Light intensity and outdoor time
(29)	Zhao Hongwei	2024	After VR training, the experimental group had statistically differences in UCVA, convergence value, negative relative accommodation and accommodation facility versus baseline and control group (*p* < 0.05).	Visual and Refractive Improvement	System	VR headset	VA, convergence value,accommodative facility, stereopsis and contrast sensitivity and asthenopia questionnaire
(20)	Janneau L. J. Claessens	2024	The home-performed VA self-assessment was able to detect subjects with VA > 0.10 logMAR with a sensitivity of 94% and a specificity of 71%.	Visual and refractive monitoring	Website	Smartphone, computer, tablet	VA and refractive error
(31)	Wu Xiaofang	2021	Mean UCVA statistically improved from 0.62 ± 0.05 to 0.84 ± 0.07 after training. The overall visual improvement rate 82.19%.	Visual and Refractive Improvement	Virtual imaging technology	VR headset	VA and ocular alignment
(21)	Qiang Su	2025	The limits of agreement corresponding to sphere, cylinder, and spherical equivalent refraction were 0.11 ± 0.89 D, −0.03 ± 0.82 D, and 0.10 ± 0.89 D in sequence.	Visual and refractive monitoring	Application	Smartphone	Refractive error
(35)	Fan Yujie	2021	The distribution of the number of people in each phase of the average daily outdoor time recorded by Akeso was ≤30 min (*n* = 16), 30 to 60 min (*n* = 26), 60 to 120 min (*n* = 4), >120 min (*n* = 0)	Environmental and behavioral assessment	Application, sensor	Wearable device	Near-work time and outdoor time
(42)	Rosa María Salmerón-Campillo	2019	Face-device distance was 36.8 ± 5.7 cm in control group and 47.2 ± 6.5 cm in intervention group. The mean total face illuminance across classrooms was recorded as 980 ± 350 lux for School #1 and 750 ± 400 lux in School #2.	Behavioral interventions	Application	Tablet	Near-work distance, light intensity
(19)	Gang Luo	2022	The SE refraction error spanned from 0 to −10.2 D. There was a small overall bias (0.17 D) in the assessment.	Visual and refractive monitoring	Application	Smartphone	Refractive error
(13)	Rajat M. Srivastava	2022	The sensitivity of photographs in identifying ophthalmic diseases spanned 81.67–95.41%, whereas the specificity spanned 86 to 99%.	Disease diagnosis	Photograph	Smartphone	Sensitivity and specificity of detecting ocular morbidities
(32)	Peng Wei	2017	The extraocular muscle training method works well for myopic children and adolescents, with a statistically improvement in visual acuity (*p* < 0.001).	Visual and Refractive Improvement	System, software	Computer, touchpad	VA
(14)	Tang Jia	2020	The deep learning model achieves the sensitivity of 0.9667 and a specificity of 0.9915 for automatic grading of myopic maculopathy and diagnosis of pathologic myopia in the high-consistency subgroup. The F1 value for lesion detection and segmentation >0.8.	Disease diagnosis	System, deep learning model	Fundus camera, web platform	Sensitivity and specificity of the system
(22)	Xiang Yixian	2022	The system showed comparable accuracy to the 5-meter standard logarithmic light box visual acuity chart (both 4.90), while inconsistent results were observed in corrected visual acuity tests.	Visual and refractive monitoring	System	Smartphone, computer	VA
(23)	Lina H. Raffa	2022	The mean difference between Snellen distance and Smart Optometry acuity was −0.023 LogMAR units or the equivalence of one letter. Smart Optometry showed a sensitivity of 89.3% and a specificity of 69.4%.	Visual and refractive monitoring	Application	Smartphone	VA
(39)	Clair A. Enthoven	2021	Continuous use consisted of 6.42 ± 4.36 uninterrupted 20-min sessions, was associated with SE of refraction and AL:CR ratio.	Environmental and behavioral assessment	Application	Smartphone	Near-work distance, outdoor time, axial length, VA, refractive error
(38)	Jun Chen	2024	Outdoor exposure lasting at least 15 min was shown to have a protective effect against myopic shift, and this protective impact takes effect only when sunlight intensity over 2000 lux.	Environmental and behavioral assessment	Light sensor, global positioning system receiver module, and pedometer	Smartwatch	Myopic shift (outdoor time and sunlight intensity)
(15)	Li Mengying	2022	The vision monitoring platform produced test results with no statistically difference from manual measurements while also reducing monitoring costs.	Disease diagnosis	System, display screen	Tablet-based vision screener	VA and refractive error
(24)	Yang Liyuan	2021	The vision warning and control system demonstrated a high detection rate of 93.3–95.8% for abnormal visual acuity, and its diagnostic results were comparable to standard hospital visual acuity assessments (*p* > 0.05).	Visual and refractive monitoring	System	Smartphone, computer	VA
(25)	Robert P. L. Wisse	2019	Refractive error between the Web-based refraction test and the professional test differed by −0.18 D for participants with myopia and 0.63 D for participants with hyperopia.	Visual and refractive monitoring	Website	Smartphone, computer	VA and refractive error
(36)	Li Liangliang	2024	Statistically significant differences (all *p* < 0.05) were observed between the myopic and non-myopic groups regarding average daily near-work time, average single near-work session duration.	Environmental and behavioral assessment	Application, sensor	Wearable device	Near-work time and distance, light intensity, outdoor time
(30)	Zihang Xu	2024	The changes in axial length and macular choroidal thickness were 0.063 ± 0.060 mm and 22.633 ± 36.171 μm in the intervention group, versus 0.129 ± 0.060 mm and 3.000 ± 31.056 μm in the control group, respectively.	Visual and refractive improvement	System	Wearable device	VA, axial length (macular choroidal thickness)

UCVA, uncorrected visual acuity; BCVA, best corrected visual acuity; LogMAR, logarithm of the minimum angle of resolution.

### 3.6 Data synthesis

To supplement the data synthesis, we performed a vote-counting analysis of studies based on the direction of effects. Results are summarized in Table 1.

Using the GRADE approach, the certainty of evidence was rated as very low for all three primary outcomes assessed (detection, treatment and management). The primary reasons for downgrading included: 1. Predominance of non-randomized and cross-sectional study designs; 2. Methodological limitations in the primary studies (12 of the 33 studies were rated Fair); 3. Substantial inconsistency across studies, particularly for refractive monitoring; 4. imprecision due to small total sample sizes for some outcomes. This very low certainty indicates that the current findings should be interpreted as preliminary, and future well-designed studies with larger sample sizes and standardized outcome measures are very likely to change the estimates (Reported effect, 95% CIs, direction of effect, and *p*-value for each included study are presented in Supplementary Table 3).

#### 3.6.1 Detection

##### 3.6.1.1 Disease diagnosis

Six studies assessed the diagnostic performance of digital tools for detecting myopia and related conditions (10–15), and were all evaluated as beneficial effects (6/6). Across the six studies, sensitivity ranged from 0.76 to 0.97, and specificity ranged from 0.76 to 1.00. Four studies reported sensitivity ≥0.80 and specificity ≥ 0.80, indicating acceptable diagnostic accuracy (10, 12–14). The highest-performing model was a deep learning system for myopic maculopathy grading (14), which achieved a sensitivity of 0.97 (95% CI: 0.912–0.989) and a specificity of 0.99 (95% CI: 0.947–1.00), with an AUC of 0.998 (95% CI: 0.995–1.000). However, two studies reported lower diagnostic performance. An AI model based on facial photos for myopia detection reported a sensitivity of 0.84 (95% CI: 0.82–0.87), specificity of 0.76 (95% CI: 0.73–0.80), and AUC of 0.84 (95% CI, 0.83–0.85) (11). Shu et al. (11) also reported that the model demonstrated better sensitivity for myopia detection in the 12–18 years age subgroup (approximately 0.85) than in the 0–5 years subgroup (0.69). Another study conducted in China observed a similar age-related pattern (15). The web-based monitoring platform showed better performance in grades 4–6 (sensitivity: 0.918) than in grades 1–3 (sensitivity: 0. 756).

##### 3.6.1.2 Visual monitoring

Across all six studies, the direction of effect consistently showed slight underestimation, with digital tools producing marginally worse visual acuity than clinical measurements (16, 20, 22–25). Visual acuity outcomes were reported using two different metrics: LogMAR and five-grade notation. Four studies reported VA bias in LogMAR units (16, 20, 23, 25), ranging from 0.02 to 0.13. The Smart Optometry application reported a bias of 0.023 LogMAR (95% CI: −0.041 to −0.006). The web-based Easee tool was evaluated in two separate studies, reporting biases of 0.02 LogMAR and 0.13 LogMAR, respectively (20, 25). However, the Netra device showed larger VA biases of 0.08 LogMAR and 0.06 LogMAR for unassisted and refined measurements (16), respectively, both of which were statistically significantly different from professional subjective refraction (*p* = 0.004 and *p* = 0.006).

The remaining two studies conducted in China reported VA bias using five-grade notation, measured with the standard 5-m light box chart (22, 24). The Wise Eye System yielded a mean bias of 0.005. Xiang et al. (22) evaluated the Wise Eye System under four testing conditions, with biases of 0.05 for monocular testing (both uncorrected and corrected) and 0 for binocular testing (both uncorrected and corrected), respectively.

##### 3.6.1.3 Refractive monitoring

Seven studies evaluated digital tools for refractive error assessment against clinical refraction (16–21, 25). Across these studies, four reported overestimation of refractive error (16–18, 20), three reported underestimation (19, 21, 25). The reported bias magnitudes ranged from 0.01 D to 1.17 D, reflecting variability across devices and testing conditions. Two studies evaluated the Netra smartphone-based tool, both reporting overestimation without modification (16, 17). Hasrod and Rubin (17) reported NETRA significantly overestimated myopia before cycloplegia, with mean differences of 1.17 D (right eye) and 1.07 D (left eye) compared with subjective refraction. After cycloplegia, the differences were reduced to 0.01 D (right eye) and 0.10 D (left eye), indicating that the overestimation was largely driven by accommodative factors. Tousignant et al. reported that unassisted Netra overestimated myopia by 0.60 D, while refined Netra showed a smaller bias of 0.1 D (16). Notably, Luo et al. (19) found that young adults in their 20s were underestimated the most (0.49 D), whereas children were overestimated (0.29 D), suggesting that age may be a significant factor influencing refractive measurement error.

#### 3.6.2 Treatment

All the seven studies (7/7) reported beneficial effects on visual acuity and/or refractive error (26–32). Improvements ranged from 0.02 to 0.29 for visual acuity and from 1.1 D to 1.99 D for refractive error. Four studies applied VR-based visual training, all achieving visual acuity improvements exceeding 0.2 (26, 27, 29, 31). Xu et al. (30) found VR-based visual training was linked to reduced axial elongation at 3 months (*p* = 0.037, mean difference = 0.038 mm) between intervention and control groups, while the significance disappeared at the 6-month follow-up (*p* = 0.172). Two studies reported refractive error outcomes. One evaluating hot moxibustion technology reported improvements ranging from 1.10 D to 1.99 D across four intervention groups (*p* < 0.01) (28), while another VR-based visual training study for amblyopia reported a refractive error improvement of 1.90 D (27).

#### 3.6.3 Management

##### 3.6.3.1 Environmental and behavioral assessment

Seven studies assessing behavioral and environmental factors did not report a directional effect and were synthesized narratively rather than through vote counting (33–39). One study assessed the accuracy of a wearable device and reported monitoring biases for near-work distance (0.54 cm), near-work duration (−3.18 min), indoor light intensity (6.50 lux), outdoor light intensity (−103.25 lux), and outdoor time (−1.33 min), all statistically significant compared with the set values (*p* < 0.001) (34). Two studies suggested that appropriate light intensity may have protective benefits for myopia, though the reported thresholds differed (6,300 lux vs. 2000 lux) (33, 38). Three studies recorded real-world behavioral data in children, with outdoor time ranging from 0.25 to 2.77 h and near-work duration ranging from 6.42 ± 4.36 episodes of 20-min to 363 min per day (35, 37, 39). One study reported that near-work duration (OR = 1.022) and single near-work session duration (OR = 1.182) were significant risk factors for myopia, while near-work distance (OR = 0.756), daytime ambient illumination (OR = 0.972), nighttime ambient illumination (OR = 0.944), outdoor activity time (OR = 0.904), and daily outdoor exposure sessions (OR = 0.439) were protective factors (36). These studies suggested associations between myopia and environmental and behavioral factors, but the long-term clinical outcomes of these factors remain to be validated.

##### 3.6.3.2 Behavioral interventions

Three studies (3/3) suggested that DHTs provided beneficial effects to correct viewing behaviors (40–42). These interventions differed from the monitoring studies by incorporating real-time alerts or screen modifications when unhealthy viewing behaviors were detected. Two studies reported specific improvements in near work distance, ranging from 1.29 to 10.4 cm (41, 42). One study applied AI notifications to behavioral modification, 65.49% of viewing distance errors and 86.48% of viewing posture errors were promptly rectified (40). The evidence suggests that active feedback-based behavioral interventions are acceptable and can possibly modify viewing behaviors. However, the small number of studies, variability in effect magnitudes, and lack of long-term follow-up preclude definitive conclusions about effectiveness.

## 4 Discussion

Asian regions face massive pressure in myopia prevention and control. In Australia, a cohort study reported that the prevalence of myopia among East Asian ethnic schoolchildren was 52.5% at age 12 and 59.1% at age 17, compared with 8.6 and 17.7% among European Caucasian children in the same age groups (43). The prevalence in Asian children, especially in the Chinese population, is higher than that in the western population (44). In a large-scale survey in Weifang, China, it was found that the city’s total myopia rate was 75.35% in school-age students (45). Starting around school age, a myopic shift and a rapid increase in myopia occur, which continue until late teenage years (46). Therefore, adolescents are in greater need of undergoing regular myopia screenings. The high prevalence of myopia in Asia, coupled with the uneven distribution of ophthalmic medical resources between urban and rural areas, highlights the urgent need for low-cost, accurate and accessible methods for myopia care. Li reported that compared with manual monitoring and management, the Internet Plus platform reduced per-student workload by 42.86% and financial expenditure by 25.97% (15). Easee supports web-based test of vision screenings at home using tablets, computers and cell phones. In accordance with the Medical Device Regulation 2017/745, the web-based test was categorized as a Conformité Européenne (CE) class 2A medical device (20). Furthermore, Ma evaluated the smartphone’s data acquisition efficiency, demonstrating that nearly 200 students could be screened per hour with only one supervising teacher alongside a consulting optometrist or nurse (10). A major limitation of this review is the geographical concentration of the evidence base: 26 of 33 studies (78.79%) were conducted in Asia. This limits the generalizability of our findings to other regions, particularly low- and middle-income countries, where differences in healthcare infrastructure, digital literacy, and device availability may affect DHT implementation. The promise of DHTs for resource-limited settings is not yet substantiated by evidence. Future research should prioritize validation in diverse geographic settings.

A key challenge in translating DHTs to pediatric myopia care is balancing their therapeutic use with established recommendations for healthy screen exposure. The methods reviewed, particularly VR-based training and smartphone-based testing, necessarily involve sustained screen engagement in children, who are already facing increasing digital media exposure at an early age in industrialized countries (47). The American Academy of Pediatrics (AAP) advises that children under 18–24 months avoid screen media (except video chatting) (48). To achieve this balance, the following principles may be considered: 1. Age-related limitations: avoid DHT use in children under 18 months where possible, and for children aged 2 to 5 years, limit screen time to 1 h per day of high-quality content (48); 2. Frequent breaks: every 20 min of device use, look at an object 20 feet away for at least 20 s (49); 3. Viewing distance: maintain a distance of approximately 25 inches from the screen (50).

Wearable devices have preliminary evidence for individuals and families to conduct environmental monitoring. At the same time, research has found that the mounting position of the wearable devices also has a certain impact on the measurement. Light intensity measured at the eye level was significantly lower than at the chest level and the wrist level. However, there was no significant difference in light exposure between the wrist level and chest level (51). This suggests that attention also needs to be paid to the location of the wearable devices if comparing the effect of light intensity on myopia in different experiments. Specific light intensity thresholds should be established for different wearable devices to distinguish indoor and outdoor environments.

The mental health status of adolescents with myopia deserves more attention, as it may be associated with quality of life and academic performance. The study has shown that myopia in children is associated with increased depressive symptoms (52). Digital technology could provide opportunities to access more mental health services and information, while also boosting patient participation and empowerment, and helping to overcome the stigma often linked to mental health services (53). Although mental health interventions were not included in this review, such approaches should be integrated into myopia care in future clinical practice.

In the promotion of DHTs, user acceptance and potential adverse reactions should not be overlooked. Professional subjective refraction was ranked first by 72% of participants and received higher comfort scores than both the unassisted and refined Netra devices across multiple domains, including distance and near vision, reading strain, image distortion, dizziness, and overall desirability of wearing the correction (16). One study reported that during virtual reality-based visual training for myopia control, children experienced virtual reality vertigo (30). Overall, some of the current DHTs still suffer from issues of poor comfort and potential safety hazards. In the future, users’ comfort and safety should be prioritized when developing a new digital method.

## 5 Limitations

First, the participants across included studies covered a broad age spectrum, including toddlers, school-aged children and adults, whereas subgroup analyses stratified by population category were not performed. Controlled studies stratified by age should be conducted in subsequent research to verify the performance of DHTs in different age subgroups. Second, sample sizes varied considerably, ranging from 23 to 6,716 subjects. The robustness of evidence generated by small-sample trials requires further validation. Third, heterogeneity existed in the outcomes, which could be attributed to the wide variety of DHTs utilized in eligible studies. Fourth, the certainty of evidence was rated as very low for all three primary outcomes (detection, treatment, and management), limiting the ability to draw firm conclusions about the effectiveness of DHTs. Lastly, the 15 cross-sectional studies can only identify correlational associations rather than causal relationships. This highlights the necessity of conducting more longitudinal studies and randomized controlled trials within this research field.

## 6 Conclusion

This systematic review evaluated DHTs in myopia care across three domains: detection, treatment, and management. For detection, DHTs show acceptable diagnostic accuracy and agreement with clinical standards for visual acuity, though visual and refractive remains systematic bias. For treatment, VR-led vision trainings have the potential to modulate visual acuity and refractive error. For management, behavioral interventions with feedback may modify unhealthy behaviors, while environmental and behavioral monitoring studies support the feasibility of DHTs. Overall, DHTs show preliminary promise across all three domains, but the evidence is limited by small sample sizes, high heterogeneity, and very low certainty across all outcomes. Well-designed, large-scale RCTs are urgently needed. Given the predominance of evidence from East Asia, applicability beyond this region remains uncertain. Future research should also address user comfort and safety.

## Data Availability

The original contributions presented in the study are included in the article/Supplementary material, further inquiries can be directed to the corresponding author.
