# Supplementary material for: Digital health technologies for myopia care: a systematic review
Source: Front Public Health. 2026 Oct 16;14:1939923. doi: 10.3389/fpubh.2026.1939923 (PMC13719632; doi:10.3389/fpubh.2026.1939923)
Supplement: Supplementary file 1 [file Table_2.docx]

**PRISMA 2020 Checklist**

| Section and Topic | Item # | Checklist item | Location where item is reported |
| --- | --- | --- | --- |
| TITLE | | |  |
| Title | 1 | Identify the report as a systematic review. | Title |
| ABSTRACT | | |  |
| Abstract | 2 | See the PRISMA 2020 for Abstracts checklist. | Abstract |
| INTRODUCTION | | |  |
| Rationale | 3 | Describe the rationale for the review in the context of existing knowledge. | Introduction |
| Objectives | 4 | Provide an explicit statement of the objective(s) or question(s) the review addresses. | Introduction |
| METHODS | | |  |
| Eligibility criteria | 5 | Specify the inclusion and exclusion criteria for the review and how studies were grouped for the syntheses. | 2.2 Inclusion and Exclusion Criteria |
| Information sources | 6 | Specify all databases, registers, websites, organisations, reference lists and other sources searched or consulted to identify studies. Specify the date when each source was last searched or consulted. | 2.1Data Sources and Search Strategy |
| Search strategy | 7 | Present the full search strategies for all databases, registers and websites, including any filters and limits used. | 2.1Data Sources and Search Strategy |
| Selection process | 8 | Specify the methods used to decide whether a study met the inclusion criteria of the review, including how many reviewers screened each record and each report retrieved, whether they worked independently, and if applicable, details of automation tools used in the process. | 2.Methods |
| Data collection process | 9 | Specify the methods used to collect data from reports, including how many reviewers collected data from each report, whether they worked independently, any processes for obtaining or confirming data from study investigators, and if applicable, details of automation tools used in the process. | 2.3 Data extraction |
| Data items | 10a | List and define all outcomes for which data were sought. Specify whether all results that were compatible with each outcome domain in each study were sought (e.g. for all measures, time points, analyses), and if not, the methods used to decide which results to collect. | 2.3 Data extraction |
|  | 10b | List and define all other variables for which data were sought (e.g. participant and intervention characteristics, funding sources). Describe any assumptions made about any missing or unclear information. | 2.3 Data extraction |
| Study risk of bias assessment | 11 | Specify the methods used to assess risk of bias in the included studies, including details of the tool(s) used, how many reviewers assessed each study and whether they worked independently, and if applicable, details of automation tools used in the process. | 2.4 Assessment of the Study Quality |
| Effect measures | 12 | Specify for each outcome the effect measure(s) (e.g. risk ratio, mean difference) used in the synthesis or presentation of results. | 2.6 Data synthesis |
| Synthesis methods | 13a | Describe the processes used to decide which studies were eligible for each synthesis (e.g. tabulating the study intervention characteristics and comparing against the planned groups for each synthesis (item #5)). | 2.6 Data synthesis |
| Reporting bias assessment | 14 | Describe any methods used to assess risk of bias due to missing results in a synthesis (arising from reporting biases). | 2.4 Assessment of the Study Quality |
| Certainty assessment | 15 | Describe any methods used to assess certainty (or confidence) in the body of evidence for an outcome. | 2.5 GRADE rating of the quality of evidence |
| RESULTS | | |  |
| Study selection | 16a | Describe the results of the search and selection process, from the number of records identified in the search to the number of studies included in the review, ideally using a flow diagram. | 3.1 Study selection |
|  | 16b | Cite studies that might appear to meet the inclusion criteria, but which were excluded, and explain why they were excluded. | Not reported |
| Study characteristics | 17 | Cite each included study and present its characteristics. | 3.2 Study characteristics |
| Risk of bias in studies | 18 | Present assessments of risk of bias for each included study. | 3.2 Study characteristics |
| Results of individual studies | 19 | For all outcomes, present, for each study: (a) summary statistics for each group (where appropriate) and (b) an effect estimate and its precision (e.g. confidence/credible interval), ideally using structured tables or plots. | From 3.3 Detection to 3.5 Management |
| Results of syntheses | 20a | For each synthesis, briefly summarise the characteristics and risk of bias among contributing studies. | 3.6 Data synthesis |
|  | 20b | Present results of all statistical syntheses conducted. If meta-analysis was done, present for each the summary estimate and its precision (e.g. confidence/credible interval) and measures of statistical heterogeneity. If comparing groups, describe the direction of the effect. | 3.6 Data synthesis |
| Reporting biases | 21 | Present assessments of risk of bias due to missing results (arising from reporting biases) for each synthesis assessed. | 3.6 Data synthesis |
| Certainty of evidence | 22 | Present assessments of certainty (or confidence) in the body of evidence for each outcome assessed. | 3.6 Data synthesis |
| DISCUSSION | | |  |
| Discussion | 23a | Provide a general interpretation of the results in the context of other evidence. | 4.Discussion |
|  | 23b | Discuss any limitations of the evidence included in the review. | 5. Limitations |
|  | 23c | Discuss any limitations of the review processes used. | 5. Limitations |
|  | 23d | Discuss implications of the results for practice, policy, and future research. | 4.Discussion |
| OTHER INFORMATION | | |  |
| Registration and protocol | 24 | Provide registration information for the review, including register name and registration number, or state that the review was not registered. | 2.1Data Sources and Search Strategy |
| Support | 25 | Describe sources of financial or non-financial support for the review, and the role of the funders or sponsors in the review. | Acknowledgements |
| Competing interests | 26 | Declare any competing interests of review authors. | Acknowledgements |
| Availability of data, code and other materials | 27 | Report which of the following are publicly available and where they can be found: template data collection forms; data extracted from included studies; data used for all analyses; analytic code; any other materials used in the review. | Acknowledgements |

**Search Strategy Overview**

**Cochrane**

#1	MeSH descriptor: [Myopia] explode all trees	1877

#2	(myopia):ti,ab,kw OR (Nearsightednesses):ti,ab,kw OR (Myopias):ti,ab,kw OR (Nearsightedness):ti,ab,kw	4329

#3	#1 or #2	4329

#4	(Artificial Intelligence):ti,ab,kw OR (digital):ti,ab,kw OR (digital health):ti,ab,kw OR (digital therapeutics):ti,ab,kw OR (Digital health intervention):ti,ab,kw	28827

#5	(Digital intervention):ti,ab,kw OR (e-health):ti,ab,kw OR (ehealth):ti,ab,kw OR (telehealth):ti,ab,kw OR (telemedicine):ti,ab,kw	25289

#6	(m-health):ti,ab,kw OR (mhealth):ti,ab,kw OR (mobile applications):ti,ab,kw OR (wearable devices):ti,ab,kw OR (wearable electronic devices):ti,ab,kw	8006

#7	(internet):ti,ab,kw OR (online systems):ti,ab,kw OR (social media):ti,ab,kw OR (virtual reality):ti,ab,kw OR (smartphone):ti,ab,kw	41037

#8	(mobile phone):ti,ab,kw OR (cellphone):ti,ab,kw OR (spectrum):ti,ab,kw OR (website):ti,ab,kw	29146

#9	#4 or #5 or #6 or #7 or #8	101273

#10	#3 and #9	120

Custom Range:01/01/2000-01/12/2025，a total of 118 trials were retrieved.

**Pubmed**

| 5 | ("artificial intelligence"[Title/Abstract] OR "digital"[Title/Abstract] OR "digital health"[Title/Abstract] OR "digital therapeutics"[Title/Abstract] OR "e-health"[Title/Abstract] OR "ehealth"[Title/Abstract] OR "telehealth"[Title/Abstract] OR "telemedicine"[Title/Abstract] OR "m-health"[Title/Abstract] OR "mhealth"[Title/Abstract] OR "mobile applications"[Title/Abstract] OR "wearable devices"[Title/Abstract] OR "wearable electronic devices"[Title/Abstract] OR "internet"[Title/Abstract] OR "online system"[Title/Abstract] OR "social media"[Title/Abstract] OR "virtual reality"[Title/Abstract] OR "smartphone"[Title/Abstract] OR "mobile phone"[Title/Abstract] OR "spectrum"[Title/Abstract] OR "website"[Title/Abstract] OR "cellphone"[Title/Abstract]) AND ("Myopia"[Title/Abstract] OR "Myopias"[Title/Abstract] OR "Nearsightedness"[Title/Abstract] OR "Myopia"[MeSH Terms]) | 975 |
| --- | --- | --- |
| 4 | ("artificial intelligence"[Title/Abstract] OR "digital"[Title/Abstract] OR "digital health"[Title/Abstract] OR "digital therapeutics"[Title/Abstract] OR "e-health"[Title/Abstract] OR "ehealth"[Title/Abstract] OR "telehealth"[Title/Abstract] OR "telemedicine"[Title/Abstract] OR "m-health"[Title/Abstract] OR "mhealth"[Title/Abstract] OR "mobile applications"[Title/Abstract] OR "wearable devices"[Title/Abstract] OR "wearable electronic devices"[Title/Abstract] OR "internet"[Title/Abstract] OR "online system"[Title/Abstract] OR "social media"[Title/Abstract] OR "virtual reality"[Title/Abstract] OR "smartphone"[Title/Abstract] OR "mobile phone"[Title/Abstract] OR "spectrum"[Title/Abstract] OR "website"[Title/Abstract] OR "cellphone"[Title/Abstract]) AND ("Myopia"[Title/Abstract] OR "Myopias"[Title/Abstract] OR "Nearsightedness"[Title/Abstract] OR "Myopia"[MeSH Terms]) | 1,012 |
| 3 | "artificial intelligence"[Title/Abstract] OR "digital"[Title/Abstract] OR "digital health"[Title/Abstract] OR "digital therapeutics"[Title/Abstract] OR "e-health"[Title/Abstract] OR "ehealth"[Title/Abstract] OR "telehealth"[Title/Abstract] OR "telemedicine"[Title/Abstract] OR "m-health"[Title/Abstract] OR "mhealth"[Title/Abstract] OR "mobile applications"[Title/Abstract] OR "wearable devices"[Title/Abstract] OR "wearable electronic devices"[Title/Abstract] OR "internet"[Title/Abstract] OR "online system"[Title/Abstract] OR "social media"[Title/Abstract] OR "virtual reality"[Title/Abstract] OR "smartphone"[Title/Abstract] OR "mobile phone"[Title/Abstract] OR "spectrum"[Title/Abstract] OR "website"[Title/Abstract] OR "cellphone"[Title/Abstract] | 1,060,966 |
| 2 | "Myopia"[Title/Abstract] OR "Myopias"[Title/Abstract] OR "Nearsightedness"[Title/Abstract] OR "Myopia"[MeSH Terms] | 33,089 |
| 1 | "Myopia"[MeSH Terms] | 23,912 |

**Embase**

| No. | Query | Results |
| --- | --- | --- |
| #6 | ('myopia'/exp OR (myopy:ab,ti OR 'near sightedness':ab,ti OR nearsightedness:ab,ti OR 'short sightedness':ab,ti OR shortsightedness:ab,ti OR 'simple myopia':ab,ti OR myopia:ab,ti)) AND ('artificial intelligence':ab,ti OR digital:ab,ti OR 'digital health':ab,ti OR 'digital therapeutics':ab,ti OR 'e health':ab,ti OR ehealth:ab,ti OR 'telehealth':ab,ti OR 'telemedicine':ab,ti OR 'm health':ab,ti OR 'mhealth':ab,ti OR 'mobile application':ab,ti OR 'wearable device':ab,ti OR 'wearable electronic devices':ab,ti OR 'internet':ab,ti OR 'online system':ab,ti OR 'social media':ab,ti OR 'virtual reality':ab,ti OR 'smartphone':ab,ti OR 'mobile phone':ab,ti OR cellphone:ab,ti OR 'spectrum':ab,ti OR website:ab,ti) AND [01-01-2000]/sd NOT [02-12-2025]/sd | 1,716 |
| #5 | ('myopia'/exp OR (myopy:ab,ti OR 'near sightedness':ab,ti OR nearsightedness:ab,ti OR 'short sightedness':ab,ti OR shortsightedness:ab,ti OR 'simple myopia':ab,ti OR myopia:ab,ti)) AND ('artificial intelligence':ab,ti OR digital:ab,ti OR 'digital health':ab,ti OR 'digital therapeutics':ab,ti OR 'e health':ab,ti OR ehealth:ab,ti OR 'telehealth':ab,ti OR 'telemedicine':ab,ti OR 'm health':ab,ti OR 'mhealth':ab,ti OR 'mobile application':ab,ti OR 'wearable device':ab,ti OR 'wearable electronic devices':ab,ti OR 'internet':ab,ti OR 'online system':ab,ti OR 'social media':ab,ti OR 'virtual reality':ab,ti OR 'smartphone':ab,ti OR 'mobile phone':ab,ti OR cellphone:ab,ti OR 'spectrum':ab,ti OR website:ab,ti) | 1,767 |
| #4 | 'artificial intelligence':ab,ti OR digital:ab,ti OR 'digital health':ab,ti OR 'digital therapeutics':ab,ti OR 'e health':ab,ti OR ehealth:ab,ti OR 'telehealth':ab,ti OR 'telemedicine':ab,ti OR 'm health':ab,ti OR 'mhealth':ab,ti OR 'mobile application':ab,ti OR 'wearable device':ab,ti OR 'wearable electronic devices':ab,ti OR 'internet':ab,ti OR 'online system':ab,ti OR 'social media':ab,ti OR 'virtual reality':ab,ti OR 'smartphone':ab,ti OR 'mobile phone':ab,ti OR cellphone:ab,ti OR 'spectrum':ab,ti OR website:ab,ti | 1,304,533 |
| #3 | 'myopia'/exp OR (myopy:ab,ti OR 'near sightedness':ab,ti OR nearsightedness:ab,ti OR 'short sightedness':ab,ti OR shortsightedness:ab,ti OR 'simple myopia':ab,ti OR myopia:ab,ti) | 46,565 |
| #2 | myopy:ab,ti OR 'near sightedness':ab,ti OR nearsightedness:ab,ti OR 'short sightedness':ab,ti OR shortsightedness:ab,ti OR 'simple myopia':ab,ti OR myopia:ab,ti | 31,222 |
| #1 | 'myopia'/exp | 39,470 |

China National Knowledge Infrastructure

SU=(myopia OR nearsightedness) AND (digitalization OR digital health OR digital therapeutics OR e-health OR telemedicine OR software OR wearable devices OR internet OR system OR social media OR virtual reality OR mobile phone OR computer OR website)

Date from 2000.01.01 to 2025.12.01, a total of 1394 trials were retrieved.

Wanfang data

M=(myopia OR nearsightedness) AND (digitalization OR digital health OR digital therapeutics OR e-health OR telemedicine OR software OR wearable devices OR internet OR system OR social media OR virtual reality OR mobile phone OR computer OR website)

Date from 2000.01.01 to 2025.12.01, a total of 701 trials were retrieved.

VIP Database

SU=(myopia OR nearsightedness) AND (digitalization OR digital health OR digital therapeutics OR e-health OR telemedicine OR software OR wearable devices OR internet OR system OR social media OR virtual reality OR mobile phone OR computer OR website)

Date from 2000.01.01 to 2025.12.01, a total of 1466 trials were retrieved.
